# Malarial Hæmoglobinuria—Answer to Dr. E. H. M. Parham

**Published:** 1891-09

**Authors:** H. M’Hatton

**Affiliations:** Macon, Ga.


### MALARIAL HÆMOGLOBINURIA—ANSWER TO DR. E. H. M. PARHAM.

BY H. M'HATTON, MACON, GA.,

I am glad that Dr. Parham accepts my etiology and symptomatology in this disease, and gives me the credit of throwing some light on the true state of the blood; from which I infer he also accepts the pathology as advanced by me. I am not aware that I said anything about quinine controlling hemorrhage, or correcting morbid secretions or eliminating effete substances. My paper was written to be read before an association where it certainly was not worth the while to go into details of the action of quinine in this disease, after the etiology and pathology were fully discussed. I said quinine is the only remedy that has any controlling effect, and it should be given in large doses. It is as near a specific as we possess in medicine in its controlling influence in all the malarial group; all other medicines can only serve us in controlling symptoms, and are consequently secondary.

It is at least reasonable to presume that I referred to the disease and not to one of its numerous symptoms, and the disease is certainly malarial. As quinine is supposed to have at least some control over malaria, I advanced it as the medicine in this condition, to prevent the destruction of the blood corpuscles. And I think at present there is no question of that action of the malarial poison. What is the use of going into a long discussion of morbid secretions and effete substances, rather indefinite terms at best, when we can reach the disease at its fountain head and prevent the formation of said secretions and substances, which are no more or less than the symptoms necessarily following the destruction of the blood corpuscles. Take for instance the every day affair of catarrhal jaundice; it certainly is better practice to endeavor to prevent the constant addition to the bile in the blood than to remove it after it is once there. Is it sound medicine to try to control this haemoglobinuria, or hemorrhage as the doctor is eroneously pleased to call it. Is it proper on the same principel to try to control the excretion of bile by the kidneys in the fore mentioned case of catarrhal jaundice. No—and unfortunately for our patients we know of no medicine that will control it.

Dr. Parham gets the natural exposatation of his malarial disease with the consequent destruction of blood corpuscles, the effect of which he recognizes as a hemorrhage, when nature begins to throw it off this so called hemorrhage grows less and less as the process of elimination goes on, which perfectly natural process, the doctor attributes to the action of his medicine.

He says "I control the hemorrhage, and alter the abnormal condition of the blood with the following: Ergotole ; Tr Iron aa. gtt x ii.

Also in his article he says "I speak from clinical experience alone." How much clinical experience has he had from ergotole, provided he got the first shipment? It was put on the market for the first time in May, 1890.

The doctor indulges in a little pleasantry at my use of the word evacuant, a good explicit word, the definition of which he can easily find. At the same time by looking up the word haemoglobinuria he will see that I deny the existence of hemorrhage, in the common sense of the term in these cases.

Some years residence in foreign countries, and the general literature of the subject, leads me to the opinion that malaria is malaria all the world over.

It is evident that the doctor does not care for statistics, also that we have plenty of cases in the south. If he can inform me where I can find a collection of twenty cases of this disease thoroughly studied prior to the publication of my paper—by any of our local men—I shall be much obliged to him. They may exist but I was not fortunate enough to find them.

I thoroughly agree with the doctor that chemical analysis is of great utility, but will do no good unless it leads to a successful course of treatment, the same proposition holding good with clinical experience.

The following statistics from Dr. Ferand of the French Marine service—and Dr. Norcum—I again publish, pending the returns from Southern Arkansas:

| | Cases. | Deaths. | Per cent | |
|---|---|---|---|---|
| a | 71 | 22 | 31 | Quinine in very small doses, calomel purgative. |
| d | 11 | 4 | 36 | Quinine in very small doses, calomel and other purgatives as the base of treatment. |
| e | 42 | 13 | 31 | |
| f | 30 | 9 | 30 | |
| b | 40 | 8 | 20 | Quinine in medium doses, calomel in small doses. |
| c | 29 | 5 | 17 | |
| g | 27 | 3 | 17 | |
| h | 18 | 2 | 11 | Quinine in large doses. |
| i | 18 | 0 | 0 | |

In the group of eighteen cases under "H," one of the deaths was due to a pernicious or siderant and the other to an intercurrent pernicious attack.

Dr. Norcum reports eleven cases with one death, which occurred one hour after he had seen the case, his treatment being large doses of quinine and hypodermics of morphia.

76 cases with 6 deaths is I think a good result in this disease.

Dr. Howell then quotes a table from Ferand showing that when quinine in very small doses is given with calomel in large doses the mortality from malaria is great, and as the quinine is increased and the calomel diminished the mortality lessens until when quinine alone is given in large doses there are no deaths at all. It is a pity Dr. Howell has not enhanced the value of his article by giving the source of his quotation, which is quite conclusive if authentic and confirmed by extended experience. What is worth doing at all is worth doing well. When will men learn that if they wish to obtain accuracy in testing a drug it must be given alone. Would that Koch could inoculate the entire medical profession with his systematic methods of investigation and dispassionate manner of estimating results.

We need in malarial hematuria as in many another foggy corner an intelligent study of cases and their careful recordings.

The facts we desire are these:

1 Does haemoglobinuria ever occur in malarial cases where no quinine, arsenic or calomel has been previously administered?

2 What is the cause of the disease if uninfluenced by treatment?

3 In what proportion of the cases do the symptoms become graver after the administration of these or other drugs given singly.

When we are supplied with such data we shall be prepared to decide this important question; until we have them all that is said on either side is of little consequence; one excellent practitioner will have a conviction that quinine cures, another an impression that it kills, and neither has taken enough pains to study the question to entitle him to have any opinion at all. Give us facts, gentlemen, and not opinions. Greater is he that can record a few cases correctly than he that hath constructed an interpretation of the apocalypse.

The above quotation is from an editorial on this subject in the Philadelphia Times and Register of April 11th 1891. In the same Journal of June 13th there is a reprint of my original article on this disease and an answer to the above questions that may interest Dr. Parham.

---

CORRECTION.

FOREST, ARK., August 11th, '91.

DEAR DOCTOR:—In my article pulished in the August No. of the Record I find the following: The bowels should be regulated by means of an emetic.—It should read: The bowels should be regulated by means of an enema.

Please make correction in your next issue,

Very Respectfully
E. H. M. Parham.

